# PEDV Structural Proteins with Emphasis on M Protein as an Immunomodulatory Factor in Porcine Innate Immunity

**DOI:** 10.3390/life16010058

**Published:** 2025-12-30

**Authors:** Jae-Yeon Park, Hye-Mi Lee

**Affiliations:** College of Veterinary Medicine, Chungnam National University, Daejeon 34134, Republic of Korea

**Keywords:** porcine epidemic diarrhea virus, membrane protein, structural proteins, innate immunity, porcine intestinal epithelium, neonatal pigs, host virus interaction

## Abstract

Porcine epidemic diarrhea virus (PEDV) is an enteric alphacoronavirus that causes severe diarrhea and high mortality in neonatal pigs, leading to substantial economic loss in the porcine industry. Previous studies have primarily focused on the spike protein because of its role in viral entry and induction of neutralizing antibody responses. However, accumulating evidence indicates that other viral components also contribute to host immune modulation and pathogenesis. This review summarizes the current knowledge on PEDV structural proteins, with an emphasis on membrane proteins as regulators of porcine innate immune responses. The molecular characteristics and intracellular localization of membrane proteins were described, and the reported effects on interferon signaling, inflammatory pathways, and cellular stress responses were examined. Findings from related coronaviruses were incorporated to highlight the conserved features and virus-specific differences in membrane protein-mediated host modulation. Available evidence suggests that membrane protein-associated interference with innate immune signaling may contribute to intestinal immune dysregulation and disease severity in neonatal piglets. The implications of these observations on PEDV pathogenesis and intervention strategies are also discussed. By shifting attention from spike-centered frameworks to structural protein-driven host interactions, this review highlights membrane proteins as an underexplored but biologically relevant factor in porcine coronavirus research.

## 1. Introduction

Porcine epidemic diarrhea virus (PEDV) is an enteric alphacoronavirus that infects swine and causes acute watery diarrhea, dehydration, and high mortality, particularly in neonatal piglets [1,2]. Since its first description, PEDV has been recognized as a major cause of economic loss in the global swine industry, with repeated outbreaks reported across Asia, Europe, and North America [3,4]. Despite extensive control efforts, PEDV continues to circulate in both endemic and epidemic forms, highlighting the limitations of current preventive strategies [5,6].

PEDV infection primarily targets the intestinal epithelium, leading to villous atrophy, impaired nutrient absorption, and disruption of intestinal barrier function [3,7,8,9]. Previous studies have demonstrated that disease severity is not solely determined by viral replication but is closely associated with host immune responses in the gut mucosa [1]. In neonatal pigs, immaturity of innate and adaptive immune mechanisms contributes to susceptibility and severe clinical outcomes [10]. These observations emphasize the importance of understanding host–virus interactions beyond viral entry and replication [11,12].

Historically, PEDV research has focused on the spike protein because of its role in receptor binding, viral entry, and induction of neutralizing antibodies [13,14]. Accordingly, vaccine development and antigenic variation studies have largely focused on spike proteins [6]. However, accumulating evidence from studies on coronaviruses indicates that other viral proteins participate in shaping host immune responses and intracellular signaling environments [12,15]. Accordingly, consideration of PEDV structural proteins is warranted to refine host–virus interaction models in the intestinal immune environment, particularly in neonatal infections [5].

In the context of the PEDV, the contribution of membrane proteins to host immune modulation remains less systematically reviewed than that of spike protein-mediated processes [1]. Although individual studies have reported interactions between PEDV proteins and innate immune signaling components, an integrated perspective focusing on structural proteins, particularly membrane proteins, is lacking [1,11,15]. Addressing this gap is essential for refining current models of PEDV pathogenesis and identifying host–virus interactions that may influence disease outcomes and intervention strategies [5].

This review focuses on PEDV structural proteins, with particular emphasis on membrane proteins as modulators of porcine innate immune responses. By summarizing the available evidence from PEDV and related coronaviruses, this review aims to provide a framework for understanding how membrane protein-mediated host interactions may contribute to intestinal immune dysregulation and disease severity in neonatal pigs.

## 2. Overview of PEDV Genome Organization and Structural Proteins

PEDV belongs to the genus Alphacoronavirus of the family *Coronaviridae* and possesses a positive-sense single-stranded RNA genome [16]. Similarly to other coronaviruses, the genome encodes a set of nonstructural proteins involved in replication and transcription, followed by genes encoding structural proteins that form the virion and participate in host–virus interactions [17]. Understanding the genomic organization of PEDV is essential to interpret the functional roles of its structural proteins in viral replication and pathogenesis.

### 2.1. General Genomic Architecture of PEDV

The PEDV genome is approximately 28 kb in length, capped, and polyadenylated, enabling direct translation upon entry into host cells [16,18]. Genomic RNA contains a large replicase gene at the 5′-prime end, composed of open reading frames 1a and 1b, which are translated into polyproteins that are subsequently processed into nonstructural proteins [16]. These nonstructural proteins form a replication-transcription complex responsible for viral RNA synthesis [17].

Downstream of the replicase gene, the genome encodes structural and accessory proteins in a conserved order characteristic of alphacoronaviruses [18]. Subgenomic mRNAs are generated through a discontinuous transcription mechanism and serve as templates for translation of structural and accessory proteins [19,20]. This genomic arrangement allows for the coordinated expression of proteins required for virion assembly, intracellular trafficking, and interactions with host cellular pathways. Overall, the genomic architecture of PEDV reflects a conserved coronavirus framework while allowing sequence variability that contributes to differences in virulence, tissue tropism, and immune modulation.

### 2.2. Structural Proteins of PEDV and Their Canonical Roles in Viral Replication

PEDV encodes four major structural proteins, spike (S), envelope (E), membrane (M), and nucleocapsid (N), each of which performs a defined role during the viral life cycle [21,22]. The S protein is a type I transmembrane glycoprotein that mediates receptor binding and membrane fusion and is the primary target of neutralizing antibodies [14,23]. Its role in viral entry has made it the central focus of vaccine development and antigenic variation studies [6,24].

E protein is a small membrane-associated protein that participates in virion assembly and bud formation [25,26]. Although present in low abundance within the virion, the envelope protein has been shown in coronaviruses to influence virion morphology and intracellular trafficking in coronaviruses [15,27]. Its contribution to viral replication efficiency is supported by studies demonstrating impaired viral production in the absence of a functional envelope protein [28,29].

The M protein is the most abundant structural protein in the virion and serves as the central organizer of viral assembly [15,30]. It interacts with other structural proteins, including spike, envelope, and nucleocapsid proteins, to coordinate virion formation within the intracellular membranes [31,32]. In addition to this canonical structural role, studies on coronaviruses have reported interactions between membrane proteins and host cellular factors, suggesting functions beyond virion assembly [33].

The N protein binds to viral RNA to form a ribonucleoprotein complex and plays a key role in genome packaging [21,34]. It has also been implicated in the regulation of viral RNA synthesis and modulation of host cell processes [35]. Together, these structural proteins enable efficient viral replication, assembly, and release while providing interfaces for host–virus interactions.

### 2.3. Summary of Conserved and Divergent Features Among Alphacoronaviruses

Comparative analyses have shown that the overall genomic organization and structural protein repertoire of PEDV are conserved among all alphacoronaviruses [14,36]. The order and general functions of the S, E, M, and N proteins are shared across this genus, reflecting common evolutionary origins and replication strategies [14,37].

Despite this conservation, sequence variation and functional divergence are evident, particularly in regions associated with host interactions and immune modulation [38,39]. Differences in spike protein sequences contribute to host range and tissue tropism, whereas variations in M and N proteins have been linked to distinct effects on host signaling pathways in different alphacoronaviruses [38,40]. These divergent features may underlie the differences in pathogenicity and immune evasion strategies observed among related viruses [38,39]. An overview of the PEDV structural proteins, including their conserved roles and reported host-interacting functions, is presented in Table 1.

In summary, PEDV structural proteins retain core functions conserved across alphacoronaviruses, while exhibiting virus-specific features that influence host interactions. This balance between conservation and divergence provides a framework for examining how individual structural proteins, particularly membrane proteins, contribute to PEDV pathogenesis and host immune modulation, which will be explored further in subsequent sections.

**Table 1:** PEDV structural proteins and their reported host-interacting functions.

PEDV Structural Protein	Canonical Role in Replication	Reported Host Interacting Function	Representative Experimental System	Ref.
S	Receptor attachment and membrane fusion during entry	Binding to sialic acids and porcine aminopeptidase N has been reported in entry studies	Cell entry assays and receptor binding studies	[41,42]
E	Virion assembly and budding	Induced ER stress and activated NF-κB, which was associated with increased IL-8 and Bcl-2 expression	Porcine intestinal epithelial cell models expressing E protein	[43]
		Blocked transcriptional activation of SLA-DR alpha and beta promoters in porcine dendritic cells, consistent with reduced MHC II expression	Bone marrow-derived dendritic cells	[44]
M	Central organizer of virion assembly via interactions with S, E, and N	Antagonized type I interferon production by targeting IRF7 function through suppression of TBK1 and IKKε, induced IRF7 phosphorylation and dimerization	HEK293T and porcine PK-15 cells with M protein expression	[45]
		Altered cell cycle-related phenotypes and increased IL-8 expression in cells expressing M protein	Cell line-based expression models	[46]
N	Viral RNA binding and genome packaging	Antagonized type I interferon production by disrupting the IRF3 and TBK1 interaction	Cell-based interferon induction assays	[47]
		Inhibited STAT1 phosphorylation and nuclear localization by promoting STAT1 acetylation through HDAC1 downregulation	Cell-based interferon signaling assays	[48]

PEDV, porcine epidemic diarrhea virus; S, spike; E, envelope; M, membrane; N, nucleocapsid; ER, endoplasmic reticulum; NF-κB, nuclear factor kappa B; IL-8, interleukin-8; TBK1, TANK-binding kinase 1; IKKε, IκB kinase epsilon; IRF, interferon regulatory factor; SLA-DR, swine leukocyte antigen-DR; MHC, major histocompatibility complex; STAT1, signal transducer and activator of transcription 1; HDAC1, histone deacetylase 1.

## 3. Structural Proteins as Active Regulators of Host Innate Immunity

Traditionally, coronavirus structural proteins have been defined by their roles in virion assembly and morphogenesis [21]. However, growing evidence across multiple coronavirus systems has demonstrated that these proteins engage in host signaling pathways that regulate innate immune responses [12,39]. This section examines the conceptual shift from purely structural functions toward immune regulatory roles, drawing on data from PEDV and other coronaviruses to frame how structural proteins contribute to host–virus interaction dynamics.

### 3.1. Shifting from Structural Roles to Immune Regulatory Functions

To interpret PEDV disease outcomes in neonatal pigs, it is necessary to consider whether structural proteins modulate innate immune signaling within intestinal cells, beyond their canonical roles in virion assembly [6,49]. This shift in perspective has practical relevance for PEDV studies because immune dysregulation in the intestinal mucosa is linked to disease severity in neonatal pigs [50]. A structural protein that attenuates interferon (IFN) induction or reshapes inflammatory signaling can plausibly affect early antiviral containment and downstream tissue injury, even if primary attention has historically been placed on spike-mediated entry and neutralizing antibody responses [14,33,43].

### 3.2. Evidence from Coronaviruses Beyond PEDV

Evidence supporting the immunoregulatory roles of structural proteins has been reported for several coronaviruses. For the M protein, studies on SARS coronavirus showed that the M protein inhibited type I IFN production by interfering with signaling complexes upstream of IFN gene transcription [51]. A related mechanism was reported for the MERS coronavirus, in which the M protein suppresses type I IFN expression and disrupts TRAF3-dependent signaling required for interferon regulatory factor (IRF) 3 activation [51,52]. Consistent observations have also been reported for SARS-CoV-2, where the M protein antagonizes mitochondrial antiviral signaling (MAVS)-mediated antiviral signaling and reduces the downstream recruitment of TRAF3, TBK1, and IRF3 [53]. Additional studies have reported that SARS-CoV-2 M protein inhibits IFN induction, including impaired IRF3 activation and reduced TBK1 signaling capacity [51].

Reviews and mechanistic studies have described the coronavirus E protein as a small membrane-associated protein with properties that extend beyond assembly, including the capacity to influence inflammatory signaling pathways [54]. In SARS coronavirus models, E protein-related determinants have been linked to enhanced inflammatory signaling, such as NF-kappa B (NF-κB) pathway activation, supporting the view that structural proteins can contribute to inflammatory phenotypes [55].

Direct interference of the nucleocapsid with IFN signaling components has been reported for PEDV and other coronaviruses [47]. In PEDV, the N protein targets TBK1 through direct interaction and impairs the association between TBK1 and IRF3, which reduces type I IFN induction [56]. Studies on other coronaviruses have similarly reported that the N protein can inhibit IFN pathway activation by disrupting the signaling steps involving TBK1 and IRF3.

### 3.3. Implications for Host Virus Interaction Studies

These findings imply that host–virus interaction studies should not treat structural proteins as purely architectural factors [12]. In contrast, experimental designs benefit from explicitly testing whether structural proteins modulate innate immune signaling outputs, including IFN induction, IFN-stimulated gene expression, and inflammatory transcriptional programs [33]. This perspective is particularly relevant for PEDV because intestinal epithelial and myeloid cell responses during early infection shape viral containment and tissue-level pathology, and structural proteins represent plausible determinants of these outcomes [6,49].

Mechanistically, available studies indicate that structural proteins frequently act at defined signaling hubs, including MAVS and TRAF3-dependent assemblies, TBK1 and IKK epsilon (IKKε) kinases, IRF activation and nuclear signaling steps [47,51]. Pathway-resolved assays that evaluate these nodes can provide interpretable readouts linking protein-specific effects to innate immune outcomes.

Taken together, evidence from PEDV and other coronaviruses supports a model in which structural proteins function as modulators of innate immune signaling rather than passive virion components. Recurrent targeting of central antiviral signaling hubs suggests that immune modulation is an evolutionarily conserved feature of coronavirus structural proteins of coronaviruses. In PEDV, this framework provides a rationale for examining membrane protein-mediated signaling in the context of porcine innate immunity and intestinal infection, and establishes a conceptual bridge to subsequent sections focusing on PEDV M protein-specific mechanisms.

## 4. PEDV M Protein Molecular Characteristics and Cellular Localization

Characterization of the molecular features and intracellular localization of PEDV M proteins is essential for interpreting their effects on host innate immune signaling. Because immunomodulatory activity is likely to be influenced by membrane topology, subcellular distribution, and interactions within the secretory pathway, the structural and trafficking properties of PEDV M protein are reviewed here as a foundation for subsequent mechanistic analyses.

### 4.1. Structural Features of PEDV M Protein

The PEDV M protein is encoded by a conserved open reading frame of approximately 681 nucleotides and produces a membrane glycoprotein of approximately 226 amino acids [57]. Sequence analyses of PEDV field isolates have shown that the M gene is relatively conserved compared to the spike gene, with most variations arising from point mutations rather than insertions or deletions [58,59]. This conservation is consistent with the essential role of the M protein during the viral life cycle and supports its use as a stable genetic marker in molecular epidemiological studies.

Topology predictions and comparative analyses with other coronaviruses indicate that the PEDV M protein contains multiple transmembrane domains and adopts canonical coronavirus M protein organization [22]. This organization includes a short luminal amino-terminal region and longer carboxy-terminal cytosolic region. This topology is compatible with the established functions of the M protein in coordinating virion assembly through interactions with other structural proteins, including S, E, and N, and engagement with host membranes [60].

In addition to its architectural role, the cytosolic domain of coronavirus M proteins has been implicated in interactions with host signaling molecules in several systems [61]. For PEDV, the conserved structural features of the M protein suggest that a similar potential interaction exists, thus providing a structural basis for the reported immunomodulatory effects observed in cell-based studies.

### 4.2. Intracellular Trafficking and Membrane Association

Coronavirus assembly occurs in the intracellular membranes within the secretory pathway, particularly in the endoplasmic reticulum, Golgi intermediate compartment, and Golgi-associated membranes [32]. Consistent with this paradigm, localization studies have demonstrated that PEDV M protein predominantly resides within secretory pathway-associated compartments when expressed in mammalian cells [61]. Co-localization with other structural proteins further supports the role of M protein in coordinating intracellular assembly sites [15,60].

Proteomic and interaction-focused analyses have identified host proteins associated with PEDV M protein, many of which are involved in membrane trafficking, vesicular transport, and intracellular organization [61]. These findings suggest that the M protein engages in the cellular machinery that governs membrane dynamics and protein distribution, which may influence both virion assembly and host cell signaling environments.

Evidence from other coronaviruses reinforces the functional importance of M-mediated trafficking [39]. In SARS-CoV-2 and related viruses, the M protein contributes to the intracellular retention of the S protein and organization of envelope components within the secretory pathway [53,62]. These observations indicate that the M protein can shape the intracellular distribution of viral proteins, thereby influencing the spatial context in which host signaling interactions occur.

### 4.3. Comparison with M Proteins of Other Coronaviruses

Comparative studies across coronaviruses have revealed that M proteins share a conserved structural architecture and are preferentially localized to secretory pathway membranes, underscoring their central role in virion assembly [15,32]. Simultaneously, virus-specific differences in targeting motifs, interaction partners, and signaling effects have been reported [15,63]. In human coronaviruses, M proteins antagonize innate immune signaling by targeting MAVS-associated complexes or downstream kinases, such as TBK1, leading to reduced activation of IRFs [51,63,64].

PEDV M protein appears to follow this general paradigm and exhibits distinct features [45]. The reported immune antagonism by the PEDV M protein primarily involves interference with IRF7-dependent type I IFN induction rather than the direct suppression of IRF3 activation, suggesting divergence in pathway node targeting. These differences may reflect adaptation to the porcine host signaling architecture and intestinal epithelial infection [6].

Taken together, structural conservation supports the shared assembly and trafficking functions of coronavirus M proteins, whereas divergence in host interaction profiles provides a basis for virus-specific immune modulation. For PEDV, integrating molecular characteristics and intracellular localization with comparative coronavirus data establishes a mechanistic framework that links the M protein structure to its role in innate immune regulation, setting the stage for a focused discussion of PEDV M protein-mediated signaling in the following section.

## 5. PEDV M Protein-Mediated Modulation of Innate Immune Signaling

Innate immune signaling is a critical determinant of early host response to PEDV infection [6]. Multiple studies have shown that PEDV interferes with antiviral IFN induction and inflammatory signaling in infected cells, and viral proteins have been implicated as mediators of this immune response [49]. This section examines how PEDV M proteins modulate key innate immune pathways, focusing on IFN signaling, inflammatory responses, and intracellular stress-associated signaling.

### 5.1. IFN Signaling Pathways

PEDV infection has been reported to suppress type I IFN responses in porcine intestinal epithelial cells and other susceptible cell types [45]. Reduced IFN beta (IFN-β) expression and impaired IFN promoter activation have been observed following viral infection or stimulation with synthetic double-stranded RNA, indicating the inhibition of antiviral signaling downstream of RNA-sensing pathways [49].

Mechanistic studies focusing on PEDV M protein have demonstrated that M expression attenuates type I IFN induction by targeting IRF7 [45]. The PEDV M protein interacts with IRF7 and inhibits TBK1- and IKKε-mediated phosphorylation and dimerization of IRF7, resulting in reduced transcriptional activation of IFN genes. Given that the PEDV M protein is a multi-spanning membrane protein with a cytosolic C-terminal domain, this region has been proposed as a likely interface for interactions with host signaling components involved in IFN regulation, although detailed domain-level mapping remains limited. These effects were associated with enhanced viral replication in cell-based systems, supporting a functional link between IFN antagonism and viral fitness.

PEDV infection has been reported to interfere with this signaling cascade at multiple levels, and PEDV M protein appears to act downstream of pattern recognition receptors (PRRs) by selectively impairing IRF7 activation rather than receptor engagement [45,49]. Importantly, these observations are largely derived from cell-based expression or infection models, and the apparent preference for IRF7 targeting may reflect the specific experimental systems used rather than a universally dominant mechanism in all PEDV-infected epithelial contexts.

### 5.2. NF-κB-Associated Inflammatory Responses

In addition to IFN antagonism, PEDV infection is associated with the activation of inflammatory signaling pathways [49]. Several studies have reported an increased expression of interleukin-8 (IL-8) in PEDV-infected porcine intestinal epithelial cells, and this induction is correlated with NF-κB activation [43,49]. The expression of individual PEDV structural proteins, including membrane and envelope proteins, is sufficient to enhance IL-8 transcription in cell-based assays, suggesting that structural proteins can directly contribute to inflammatory gene regulation [43,46].

IL-8 is a key chemokine involved in the recruitment and activation of immune cells at mucosal surfaces [65]. Increased IL-8 production by the infected epithelial cells may promote local inflammatory cell infiltration and shape the intestinal immune environment during PEDV infection [49,66]. Although the direct effects of PEDV M protein on myeloid cells remain less extensively characterized, epithelial-derived inflammatory cues are likely to influence downstream activation of innate immune cells in the gut [6].

### 5.3. Endoplasmic Reticulum (ER) Stress and Intracellular Signaling Crosstalk

PEDV infection has been associated with the activation of endoplasmic reticulum stress and unfolded protein response pathways in porcine intestinal tissues and cultured cells [49]. Increased expression of ER stress markers and activation of UPR-related signaling components have been reported in infected epithelial cells and intestinal samples, often accompanied by apoptotic responses [43]. These observations indicate that PEDV infection disrupts cellular homeostasis by intersecting innate immune regulation.

The PEDV E protein induces ER stress and activates UPR-associated signaling pathways in infected cells [43]. In contrast, direct evidence linking the PEDV M protein to ER stress induction is currently lacking, although its localization within the secretory pathway suggests potential indirect effects on the ER. Nevertheless, given the localization of the M protein to secretory pathway membranes, indirect effects on ER homeostasis and stress-responsive signaling cannot be excluded [61]. Accordingly, current evidence supports a model in which ER stress during PEDV infection is driven predominantly by the E protein, whereas the contribution of the M protein, if any, is likely indirect and context dependent.

ER stress and UPR signaling intersect with innate immune pathways by influencing translation, inflammatory gene expression, and cell survival [67]. In the context of PEDV infection, IFN and inflammatory signaling are modulated within this broad intracellular stress environment [43,49]. Within this framework, the PEDV M protein appears to primarily suppress antiviral IFN responses, whereas ER stress and inflammatory signaling are largely attributed to E protein activity, together suggesting a potential functional division of labor among the PEDV structural proteins. The reported host signaling pathways targeted by the PEDV M protein and associated experimental evidence are summarized in Table 2. Taken together, the available evidence indicates that the PEDV M protein modulates innate immune signaling primarily by interfering with type I IFN induction, whereas inflammatory and stress-related pathways provide an important contextual framework that shapes overall host responses during infection.

## 6. Comparison of PEDV M Protein with M Proteins of Other Coronaviruses

Comparative analyses of coronavirus membrane proteins have provided important insights into the conserved and virus-specific mechanisms by which these proteins influence the host innate immune responses. Although the PEDV M protein has been less extensively characterized than the M proteins from human coronaviruses, the available data allow the contextualization of PEDV M within broader coronavirus biology and help to define features that are likely to be shared or distinct.

### 6.1. Shared Immune Modulatory Strategies

Studies on multiple coronaviruses have shown that M proteins commonly target the central nodes of innate immune signaling rather than upstream PRRs [12,64]. In SARS coronavirus, the M protein interferes with type I IFN induction by disrupting the signaling complexes required for IRF activation [64]. Similar effects were reported for the MERS coronavirus, in which the M protein suppressed IFN production by interfering with adaptor and kinase assemblies downstream of RNA-sensing pathways [42]. More recently, SARS-CoV-2 M protein has been shown to antagonize MAVS-dependent signaling by impairing the recruitment of TRAF3, TBK1, and IRF3, resulting in reduced IFN responses [53,63].

**Figure 1:**
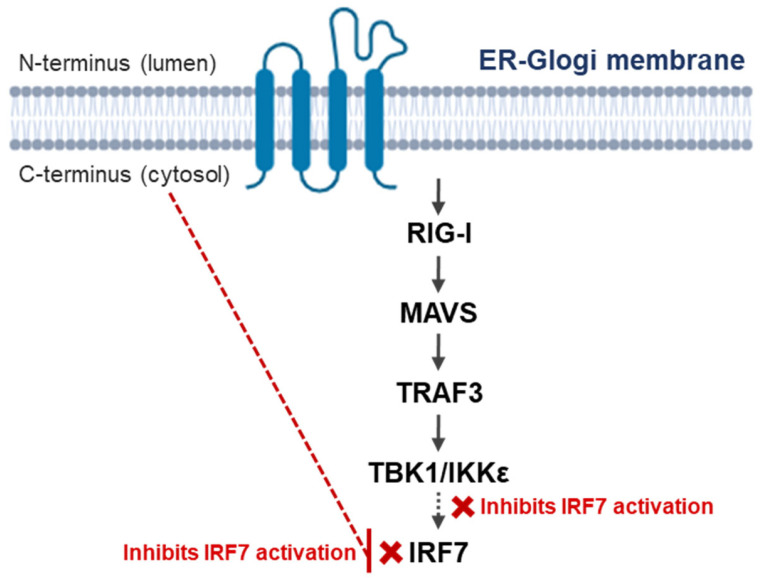
Integrated schematic of PEDV M protein topology and interference with innate immune signaling. The porcine epidemic diarrhea virus membrane protein localizes to the ER–Golgi membrane and adopts a multi-pass transmembrane topology with its C-terminus exposed to the cytosol. The cytosolic C-terminus interferes with the RIG-I–MAVS–TRAF3 signaling cascade by inhibiting TBK1 and IKKε-mediated activation of IRF7, thereby suppressing downstream type-I interferon signaling. Solid arrows indicate canonical signaling progression, whereas dashed red lines and red crosses indicate the inhibitory effects of the PEDV M protein on IRF7 activation.

These observations indicate that coronavirus M proteins frequently converge on the MAVS–TBK1–IRF axis to attenuate antiviral signaling [42,53,64]. The PEDV M protein fits within this conserved framework, supporting the view that targeting downstream antiviral signaling hubs is a shared strategy among coronaviruses [49,70]. An integrated schematic summarizing PEDV M protein topology and its interference with this signaling axis is shown in Figure 1. The recurrent targeting of shared signaling hubs suggests that immune modulation by M proteins represents a conserved functional layer superimposed on the structural role of these proteins in virion assembly.

### 6.2. Virus-Specific Adaptations in PEDV

Despite these shared strategies, virus-specific adaptations are evident in the interactions between individual M proteins and host signaling pathways [52,64]. In PEDV, the reported immune antagonism by M protein is primarily linked to interference with IRF7-mediated type I IFN induction, whereas the effects on IRF3 appear less prominent in the current literature [47]. This is in contrast to several human coronaviruses, in which M protein activity has been described at the level of IRF3 activation or MAVS complex formation [53,64].

In addition, PEDV M protein studies have largely been conducted in porcine or heterologous cell systems using ectopic expression, and direct in vivo validation remains limited [49,70]. These experimental contexts may influence the apparent specificity of signaling targets. Sequence conservation within the PEDV M protein suggests evolutionary pressure to maintain core functions. However, subtle differences in the cytosolic domains or interaction motifs may account for distinct host-protein interactions in porcine cells [2,45,70]. Such adaptations are likely shaped by tissue tropism in the intestinal epithelium and immune landscape of neonatal pigs.

### 6.3. Lessons from SARS-CoV, MERS-CoV, and Related Animal Coronaviruses

Mechanistic studies of SARS coronavirus, MERS coronavirus, and SARS-CoV-2 have demonstrated that M proteins can serve as scaffolds that reorganize signaling complexes in intracellular membranes [42,53,64]. These studies have benefited from integrated approaches combining localization analysis, protein interaction mapping, and pathway-specific functional assays. A comparative overview of the immunomodulatory roles of PEDV and other coronavirus M proteins is presented in Table 3. Applying similar strategies to the PEDV M protein would facilitate a more precise understanding of its immunomodulatory mechanisms in porcine models.

Comparative data from animal coronaviruses further highlight the importance of the host species [72]. Differences in adaptor usage, IRF expression, and cell type-specific signaling thresholds may influence the manifestation of M protein-mediated antagonism in vivo [73,74]. Lessons from these systems underscore the need to evaluate PEDV M protein function in primary porcine intestinal epithelial and immune cells, where the signaling architecture more closely reflects physiological conditions [6,49].

Taken together, a comparison with other coronaviruses indicates that the PEDV M protein likely employs conserved immunomodulatory principles, while exhibiting adaptations shaped by host species and tissue tropism. These insights provide a conceptual framework for interpreting existing PEDV data and guide future studies aimed at resolving the contribution of M protein to porcine innate immune regulation and disease pathogenesis.

**Table 3:** Comparison of immune modulatory roles of coronavirus M proteins.

Coronavirus	Genus	Reported Immune Modulatory Role of M Protein	Primary Pathway Node or Step Affected	Experimental Context Reported	Ref.
PEDV	Alphacoronavirus	Suppressed type I IFN induction	IRF7 activation, reduced TBK1 and IKKε driven IRF7 phosphorylation and dimerization	M protein expression in mammalian cells, including porcine cells	[45]
SARS-CoV-2	Betacoronavirus	Inhibited type I IFN gene transcription	Impeded formation of the TRAF3-containing signaling complex required for IFN induction	M protein expression and reporter assays	[64]
		IFN antagonism mapped to M protein transmembrane domain 1	IFN induction pathway, region mapping linked to Golgi targeting and IFN antagonism	Comparative analysis of SARS coronavirus M and HKU1 M	[64]
		Negative regulator of innate antiviral response	Interaction with MAVS and impaired recruitment of TRAF3, TBK1 IRF3 to the MAVS complex	M protein expression with signaling assays and interaction studies	[63]
		Suppressed IFN-β and ISG induction by promoting TBK1 degradation	K48 linked ubiquitination and degradation of TBK1, impaired TRAF3 TANK TBK1 IKKε complex formation	M protein expression and reporter assays	[51]
		Inhibited type I and type III IFN induction by disrupting RIG-I MAVS axis	Reduced formation of RIG I MAVS, MAVS TBK1, and TRAF3 TBK1 interactions, reduced IRF3 phosphorylation and nuclear translocation	M protein expression with pathway stimulation and co-immunoprecipitation	[53]
		Suppressed type I IFN expression, the effect described as IRF3 specific	Interaction with TRAF3 and disruption of TRAF3 TBK1 association, reduced IRF3 activation	M protein expression in HEK 293 cells with poly I:C or Sendai virus stimulation	[52]
Human coronavirus HKU1	Betacoronavirus	No detectable inhibition of IFN production in the comparative system	No inhibitory signaling node identified	Comparative analysis with SARS coronavirus M	[75]
Human coronavirus OC43	Betacoronavirus	Reduced transcriptional activation of antiviral response elements	Reduced ISRE, IFN-β promoter, and NF-κB response element activity in reporter assays	M protein expression in HEK 293 cells with Sendai virus, IFN alpha, or TNF alpha stimulation	[76]

CoV, coronavirus; PEDV, porcine epidemic diarrhea virus; IFN, interferon; ISG, interferon-stimulated gene; IRF, interferon regulatory factor; RIG I, retinoic acid-inducible gene I; MAVS, mitochondrial antiviral signaling protein; TRAF3, TNF receptor-associated factor 3; TBK1, TANK-binding kinase 1; IKKε, IKK epsilon; NF-κB, nuclear factor-kappa B.

## 7. Implications for Disease Pathogenesis in Neonatal Pigs

Building on comparative insights from M biology, understanding how PEDV M protein-mediated signaling interfaces with host innate immunity is particularly relevant in neonatal pigs, in which intestinal immune responses are developmentally constrained. This section integrates epithelial-centered innate immune regulation with reported M protein functions to frame disease severity and age-dependent susceptibility.

### 7.1. Innate Immune Dysregulation in the Intestinal Epithelium

PEDV primarily infects differentiated enterocytes lining the small intestine, and epithelial innate immune responses constitute the first layer of antiviral defense [50,77,78]. In neonatal pigs, these responses are characterized by limited basal IFN production, delayed induction of IFN-stimulated genes, and reduced functional redundancy among pattern recognition pathways [79,80,81]. Experimental and field studies have shown that PEDV infection is associated with blunted type I IFN responses in intestinal epithelial cells despite robust viral replication, indicating ineffective early antiviral signaling [68,82].

In parallel, PEDV infection has been linked to the induction of epithelial inflammatory mediators such as IL-8, which can reshape the local immune environment without necessarily promoting effective viral clearance [82,83,84]. This combination of insufficient antiviral IFN signaling and selective inflammatory activation represents a form of innate immune dysregulation that favors viral persistence and contributes to epithelial damage and villous atrophy [77,78,85]. Such epithelial-level imbalances provide a permissive context in which viral immune antagonists exert disproportionate effects on disease outcomes [68,86].

### 7.2. Contribution of M Protein-Mediated Signaling to Disease Severity

Within this epithelial context, PEDV M protein-mediated suppression of IFN signaling influences disease severity [70]. By impairing IRF7-dependent IFN induction downstream of RNA-sensing pathways, the M protein can reduce the magnitude and timing of antiviral gene expression in infected epithelial cells. Delayed or insufficient IFN responses are associated with higher viral loads and prolonged epithelial infections, both of which exacerbate tissue injury [50,87].

Simultaneously, the reported associations between PEDV structural proteins and NF-κB-dependent inflammatory responses suggest that M protein activity may coexist with selective induction of chemokines such as IL-8 [83]. This imbalance, characterized by attenuated antiviral defense along with preserved or enhanced inflammatory signaling, may contribute to immune-mediated tissue pathology rather than to efficient viral control [1,83]. Although direct in vivo evidence linking M protein function to disease severity remains limited, the available mechanistic data support a model in which M protein-mediated signaling shifts epithelial responses toward a pathogenic profile.

### 7.3. Relevance to Age-Dependent Susceptibility

Age-dependent susceptibility is a defining feature of PEDV pathogenesis, with neonatal pigs experiencing more severe disease and higher mortality rates than older animals [88]. This difference is closely linked to the developmental immaturity of innate immune pathways in the neonatal intestine, including reduced IFN competence and altered epithelial signaling thresholds. In this context, viral proteins that antagonize innate immunity are likely to have amplified effects [1].

Therefore, PEDV M protein-mediated interference with IFN signaling may be particularly consequential in neonatal hosts, where compensatory antiviral mechanisms are limited [1]. As immune maturation progresses with age, redundancy within innate signaling networks may partially offset viral antagonism and reduce disease severity [10]. This framework aligns with observations that similar viral exposures produce markedly different clinical outcomes, depending on the age of the host.

Collectively, epithelial innate immune dysregulation, M protein-mediated modulation of antiviral signaling, and developmental constraints in neonatal pigs form an integrated model of PEDV pathogenesis in neonatal pigs. This perspective emphasizes host–virus interaction dynamics rather than viral replication alone as the key determinant of disease severity in neonatal infections.

## 8. Implications for Vaccine and Antiviral Research

Mechanistic insights into PEDV M protein-mediated modulation of innate immune signaling extend beyond pathogenesis to the design and interpretation of intervention strategies. Because vaccine efficacy and antiviral outcomes are influenced not only by antigen-specific immunity but also by the innate signaling environment in which immune responses are initiated, understanding how PEDV proteins shape this context is relevant to translational research. This section discusses how current PEDV intervention strategies align with the emerging knowledge of viral immune modulation and highlights the considerations for incorporating the immune context into future vaccine and antiviral studies.

### 8.1. Why M Protein Underrepresented in Current Vaccine Strategies

Most PEDV vaccine developments have prioritized the spike protein because it contains key neutralizing epitopes and functions in receptor binding and entry, which are directly linked to protective antibody responses [13]. This focus is reflected across diverse vaccine platforms, including protein subunits, nanoparticle formulations, mRNA vaccines, and epitope-based designs that center on S or S1 as the main immunogens [89,90].

In contrast, the M protein has typically been treated as a structural scaffold that supports assembly rather than as a leading immunogen for protective immunity [91]. The second factor is the practical immunogenicity. Neutralization assays and correlates of protection in PEDV commonly map to spike-directed antibodies, whereas robust protection by M-focused antibody responses has not yet been established as the dominant mechanism [92,93]. Although studies have begun to explore M epitopes and M-related immune responses, these efforts remain limited compared to spike-centered work.

Risk management is the third factor in product development. Vaccine pipelines often select antigens with established protective readouts and standardized assays, which favor spike proteins, because neutralizing activity can be measured and benchmarked across strains and platforms [94]. These considerations collectively explain why M has been underrepresented in current PEDV vaccine strategies, despite the increasing mechanistic interest in M-driven host modulation.

### 8.2. Potential Value of Incorporating Immune Context Shaping Factors

Mechanistic studies have shown that PEDV encodes proteins that suppress IFN induction and reshape innate immune signaling, supporting a model in which immune evasion contributes to replication efficiency and pathogenesis [70]. Within this framework, PEDV M protein has been reported to interacts with IRF7 and inhibits type I IFN production by impairing TBK1- and IKKε-mediated IRF7 activation [45]. This type of activity positions M as an immune context-shaping factor rather than a classical protective antigen, because altering IFN competence can influence early antiviral state formation and downstream inflammatory balance [95]. Importantly, this conceptual role does not imply that M protein is a practical target for antibody-based vaccination, given its intracellular localization and lack of evidence for neutralizing antibody efficacy.

From a vaccine and intervention perspective, immune context-shaping factors are important because protective outcomes depend not only on antigen-specific responses, but also on the innate signaling environment that supports mucosal immunity [70]. Spike-based vaccination can induce neutralizing antibodies; however, infection outcomes can still be influenced by viral strategies that blunt IFN signaling or manipulate inflammatory programs [68]. Incorporating this concept into PEDV research encourages the evaluation of interventions that preserve or restore antiviral signaling in the intestinal epithelium and immune cells, including approaches that support type I or III IFN activity during early infection [96,97].

Accordingly, the translational relevance of M protein-mediated immune modulation may lie more in informing adjuvant selection, immunization routes, and complementary antiviral strategies than in serving as a primary vaccine antigen. In this context, the relatively conserved nature of the M protein also suggests its potential relevance for T cell-directed immune responses rather than antibody-mediated neutralization.

### 8.3. Considerations for Future PEDV Intervention Studies

Future PEDV intervention studies will benefit from designs that evaluate both antigen-specific immunity and innate pathway competence in porcine models. As PEDV primarily targets the small intestinal epithelium, outcomes should be assessed using measurements aligned with mucosal protection, including IgA responses, viral shedding, and epithelial IFN-stimulated gene induction in intestinal tissues [3,98,99]. Neonatal susceptibility also supports age-stratified models, since innate immune baselines differ substantially between suckling piglets and older pigs [95].

For M protein-specific questions, priority should be placed on separating the effects observed under ectopic expression from the effects during infection, and validating pathway targets in primary porcine intestinal epithelial cells and myeloid populations [1]. The reported M IRF7 interaction provides a defined hypothesis that can be tested using pathway-resolved assays that track TBK1 and IKKε activity, IRF7 activation state, and downstream IFN outputs [45]. In parallel, the integration of host-directed strategies may be appropriate when viral resistance to IFN signaling has been documented in infection models [95].

Overall, vaccine and antiviral development for PEDV is likely to advance when spike-focused neutralization strategies are paired with a systematic evaluation of viral innate immune antagonism, including the contribution of M protein-mediated signaling in age-appropriate porcine models and intestinal tissue contexts.

## 9. Knowledge Gaps and Future Research Directions

Despite growing mechanistic insights into PEDV M protein-mediated modulation of innate immune signaling, important gaps remain in the current literature that limit the integration of these findings into models of disease pathogenesis and intervention. Many conclusions have been drawn from reductionist experimental systems, and the extent to which the reported molecular interactions occur during intestinal infection in neonatal pigs remains unclear. This section outlines the key experimental and conceptual limitations of existing studies and identifies priorities for future research aimed at resolving the contribution of the PEDV M protein to host–virus interactions in physiologically relevant contexts.

### 9.1. Experimental Limitations in Current PEDV M Protein Studies

Most mechanistic studies of the PEDV M protein have used ectopic expression in transformed cell lines, together with IFN reporter assays and stimulated signaling readouts [45]. These approaches identified IRF7 as a target of the PEDV M protein and showed reduced TBK1- and IKKε-driven IRF7 phosphorylation and dimerization, with decreased type I IFN output. While these findings provide a clear molecular hypothesis, several limitations should be considered when interpreting their relevance to intestinal infections [100]. Overexpression can alter membrane distribution, stoichiometry with other viral proteins, and access to signaling complexes, which can affect the apparent pathway specificity. In addition, many IFN antagonism studies have been conducted in non-intestinal or non-porcine lines, which may differ from the porcine intestinal epithelium in terms of baseline IFN competence and IRF usage.

Second, PEDV infection studies have documented broad suppression of dsRNA-induced IFN-β responses through RIG-I-mediated signaling blockade, indicating that multiple viral factors act in parallel [68]. This makes it difficult to assign the magnitude of immune suppression to the M protein alone without infection context experiments that preserve the timing and subcellular organization of viral protein expression.

Third, inflammatory signaling outputs have been linked to PEDV infection in the intestinal epithelial systems, including NF-κB activation and IL-8 induction. A recent study reported that the expression of PEDV M or E proteins alone was sufficient to increase IL-8 levels in cell-based assays [68]. However, mechanistic mapping of the M protein to specific NF-κB signaling nodes remains limited compared to the IFN-focused IRF7 mechanism [101].

### 9.2. Need for In Vivo and Primary Porcine Cell-Based Analyses

Progress in defining PEDV M protein function likely depends on validation in primary porcine intestinal epithelial cells and relevant porcine innate immune cell populations, because PEDV disease is driven by intestinal epithelial infection and mucosal immune responses [102]. Infection-based studies in porcine intestinal epithelial models have shown that PEDV blocks dsRNA-induced IFN-β production through RIG-I-mediated signaling interference, supporting the need to evaluate the effects of M proteins under infection conditions and within intestinal cell types [69]. Importantly, future studies should incorporate longitudinal analyses that correlate M protein-mediated modulation of IFN signaling with viral replication dynamics, epithelial injury, and disease severity over the course of infection.

In vivo or ex vivo intestinal tissue studies are important because epithelial stress signaling and local cytokine networks can reshape innate signaling baselines and influence pathway readouts [1]. In addition, strain-dependent differences have been reported in PEDV-induced type I and type III IFN outputs and in the involvement of RIG-I-like receptors and TLR pathways, which supports the evaluation of representative strains when testing M protein-mediated effects. Given the central role of type III IFN in intestinal antiviral defense, defining how M protein activity intersects with IFN lambda signaling in vivo represents a key unresolved question.

A practical approach involves the use of reverse genetics to generate viruses with targeted M mutations that disrupt the IRF7 interaction interface described in cell-based studies, followed by phenotyping in primary intestinal epithelial cultures and neonatal pig infection models [1]. Such studies could link the molecular mechanisms of viral replication, IFN kinetics, inflammatory mediator profiles, and clinical outcomes.

### 9.3. Relevance to Broader Coronavirus Biology

Studies on human coronaviruses have established that M proteins can function as innate immune antagonists by targeting central adaptor and kinase assemblies such as MAVS, TRAF3, and TBK1, leading to reduced IFN induction [63]. These findings support the concept that immune modulation is a conserved layer of M protein function across coronaviruses, with differences in pathway node targeting and host factor usage across viruses [52,63].

PEDV M protein targeting by IRF7 extends this comparative framework by highlighting IRF-specific modulation that may be shaped by host species and tissue context [45]. Integrating PEDV M biology with the broader coronavirus literature will help to define the shared principles of membrane-anchored immune antagonism and identify features that reflect alphacoronavirus adaptation to intestinal infection in neonatal hosts.

Taken together, current evidence supports a mechanistic model for PEDV M protein-mediated IFN antagonism. However, this field still requires infection context validation, primary porcine cell testing, and in vivo evaluation to establish the contribution of M protein-mediated signaling to PEDV disease phenotypes and general principles of coronavirus immune modulation.

## 10. Conclusions

PEDV pathogenesis is increasingly recognized as a process shaped not only by viral replication efficiency but also by the quality and timing of host innate immune responses, particularly in neonatal pigs. Although spike protein-mediated entry and antibody neutralization have provided a foundational framework for PEDV research and vaccine development, this focus alone does not fully account for immune dysregulation and disease severity observed in susceptible hosts.

The evidence reviewed here supports the concept that coronavirus structural proteins function as active determinants of host immune outcomes rather than as passive components of the virion architecture. In PEDV, the membrane protein exemplifies this principle by modulating key innate immune signaling pathways downstream of PRRs. The reported interference with IRF7-dependent type I IFN induction places the PEDV M protein within a conserved coronavirus strategy of targeting central antiviral signaling nodes, while also revealing virus-specific adaptations shaped by porcine host biology.

Importantly, PEDV M protein-mediated immune modulation occurs in the context of the developmental immaturity of the neonatal intestinal immune system. In this setting, the suppression of antiviral IFN responses combined with selective inflammatory signaling may shift epithelial responses toward a pathogenic profile that favors viral persistence and tissue injury. This framework provides a mechanistic explanation for age-dependent disease severity that extends beyond differences in viral exposure or replication.

Beyond PEDV-specific implications, this review emphasizes the broader value of examining structural proteins as contributors to immune regulation during coronavirus infections. Focusing on membrane protein-mediated host interactions highlights the importance of evaluating viral proteins within defined cellular and developmental contexts rather than as isolated molecular entities. This approach encourages the integration of virological, immunological, and host developmental perspectives when interpreting the disease mechanisms.

By reframing PEDV pathogenesis through the lens of structural protein-driven immune modulation, this review provides a conceptual basis for future studies aimed at refining intervention strategies and experimental designs. Incorporating the immune context and host–virus interaction dynamics along with spike-centered approaches may improve our understanding of PEDV disease outcomes and inform more comprehensive strategies for virus control in neonatal pigs.

## Figures and Tables

**Table 2:** Reported host signaling pathways modulated by PEDV M protein.

Host Signaling Pathway	Level of Modulation	Reported Molecular Target or Mechanism	Experimental System	Ref.
Type I IFN signaling	Suppression of IFN induction	Interaction with IRF7 leading to inhibition of TBK1 and IKKε-mediated phosphorylation and dimerization of IRF7	HEK293T cells and porcine PK-15 cells expressing PEDV M protein	[45,68]
RIG I like receptor downstream signaling	Inhibition of PRR downstream signaling	Impaired activation of IRF7 downstream of MAVS without direct interference at the receptor level	Cell-based IFN reporter assays	[45,47]
IFN-β promoter activation	Reduced transcriptional activity	Decreased IFN-β promoter-driven luciferase activity upon M protein expression	Luciferase reporter assays in mammalian cells	[45,69]
NF-κB-associated inflammatory signaling	Induction of inflammatory chemokine expression	Increased IL-8 transcription associated with NF-κB activation in cells expressing PEDV structural proteins, including M	Porcine intestinal epithelial cell models	[43,70]
Cell cycle-associated signaling	Alteration of cell cycle progression	Changes in cell cycle distribution and increased IL-8 expression linked to M protein expression	Mammalian cell lines transfected with PEDV M protein	[46]
Secretory pathway-associated signaling	Indirect modulation	Localization of M protein to secretory pathway membranes, suggesting potential influence on signaling hubs linked to ER Golgi compartments	Immunofluorescence and localization studies	[46,71]

PEDV, porcine epidemic diarrhea virus; IFN, interferon; IRF7, interferon regulatory factor 7; TBK1, TANK-binding kinase 1; IKKε, IκB kinase epsilon; NF-κB, nuclear factor kappa B; IFN-β, IFN beta; RIG I, retinoic acid-inducible gene I; MDA5, melanoma differentiation-associated protein 5; MAVS, mitochondrial antiviral signaling protein; PRR, pattern recognition receptor; UPR, unfolded protein response; ER, endoplasmic reticulum.

## Data Availability

No new data were created or analyzed in this study.
